# Assessing the impact of HIV self-testing on diagnosis rates in vulnerable groups in belo horizonte, Brazil: A cross-sectional analysis

**DOI:** 10.1016/j.puhip.2024.100567

**Published:** 2024-12-22

**Authors:** João Vitor Innecco Arêas, Gabriela Bragança Costa e Moreira, Gabriela Picchioni Baêta, João Vitor Levindo Coelho Novaes, Luísa Castro de Sousa Pires, Luara Isabela dos Santos

**Affiliations:** Faculdade Ciências Médicas de Minas Gerais, Belo Horizonte, Minas Gerais, Brazil

**Keywords:** HIV, HIV testing, Brazil, Developing countries, Health risk behaviors, Sexually transmitted infections

## Abstract

**Background:**

In recent years, HIV self-testing (HIVST) has emerged as a promising approach to enhance accessibility and uptake of HIV testing, particularly among populations at high risk for sexually transmitted infections (STIs). Despite its potential benefits, the effectiveness, and challenges of HIVST warrant careful examination to inform public health strategies effectively. This study investigates the effectiveness and challenges of HIV self-testing (HIVST) in populations at high risk for sexually transmitted infections (STIs). **Study design**: Cross-sectional study.

**Methods:**

We employed questionnaires, HIVST, and standard tests for HIV, Hepatitis C, and Syphilis, in patients exhibiting STI risk behaviors in Belo Horizonte, Brazil, between August and November of 2019.

**Results:**

We engaged 125 individuals, median age of 33.5 years, with most participants (61 %) deviating significantly during self-testing. Despite this, HIVST was generally perceived as user-friendly. From the perspective of health professionals, there was 100 % agreement between HIVST and the gold standard HIV testing results. Notably, among those seeking solely HIV testing, 19.2 % tested positive for Syphilis, and 4.8 % for Hepatitis C. Only a minority (4.8 %) were aware of the HIV window period.

**Conclusion:**

While HIVST presents benefits, the evidence does not yet support its widespread adoption as a standalone public health policy. Moreover, exclusive reliance on HIVST might mask the prevalence of other STIs. We advocate for a holistic approach to HIV and STI testing, incorporating education, counseling, and comprehensive healthcare access in public health initiatives.

## Graphical abstract

**Figure:**
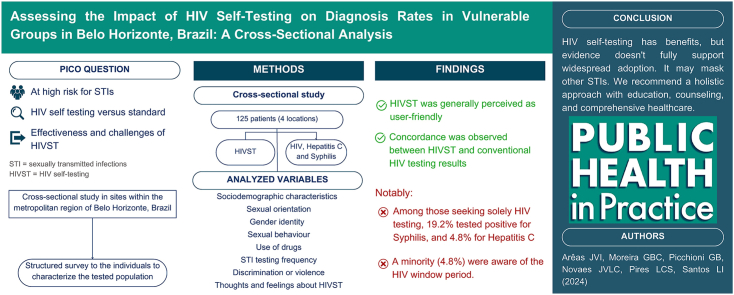

## 1 Introduction

The isolation of the Human Immunodeficiency Virus (HIV) occurred more than two decades ago. However, the epidemiological control of this infection remains a public health concern. At the end of 2023, the number of people living with HIV reached 39.9 million, with the vast majority in low- and middle-income countries. However, only 30.7 million had access to antiretroviral therapy, and 630,000 people died from AIDS-related diseases that year [1]. In Brazil, between 2020 and 2022, HIV infections increased by 17.2 %, with a mortality rate of 4.1 per 100,000 inhabitants by the end of 2022 [2]. Like other undeveloped nations, Brazil suffers from social inequality, poverty, precarious work, housing, and education conditions, and these factors are relevant to the rate of HIV infection [3,4].

Syphilis is a curable bacterial sexually transmitted infection (STI) caused by *Treponema pallidum*. It is preventable through safe sex practices and screening in selected populations, with particular concern regarding mother-to-child transmission. Since most infections are asymptomatic or go unrecognized, approximately 8 million adults aged 15–49 years worldwide acquired syphilis in 2022 [5]. In Brazil, between 2012 and 2022, 1,237,027 cases of acquired syphilis, 537,401 cases of syphilis in pregnant women, 238,387 cases of congenital syphilis, and 2153 deaths due to congenital syphilis were reported [6].

Viral hepatitis is one of the communicable diseases with increasing deaths, with an estimated number rising from 1.1 million in 2019 to 1.3 million in 2022 around the globe. Hepatitis C caused 17 % of these deaths, and there is evidence suggesting that hepatitis-related cancer cases and deaths are increasing. Of the 2.2 million new infections, 1 million were caused by the hepatitis C virus (HCV) [7]. Hepatitis C was the leading cause of death from viral hepatitis in Brazil, and, between 2000 and 2023, 318,916 cases were confirmed. Of these cases, 7.8 % presented with HIV coinfection, with a variation observed from 8.6 % in 2014 to 7.6 % in 2023 [8].

According to the Joint United Nations Program on HIV/AIDS, people from key populations include sex workers, men who have sex with men (MSM), people who inject drugs, transgender people, and people in prisons and other closed settings [9]. These key populations have limited access to prevention, medical care, and treatment services because they are often stigmatized and even criminalized [10]. There is an urgency to ensure that these populations are fully included in public policies, and thus, testing services must be available to them to facilitate immediate treatment and more optimistic results in controlling the infection rate [[11], [12], [13]].

Available HIV tests include the detection of antibodies, antigens, or nucleic acids. Most rapid tests approved by the U.S. Food and Drug Administration (FDA) are antibody tests [14]. There are also antigen/antibody immunoassays, capable of detecting HIV-1 and HIV-2 antibodies as well as the HIV-1 p24 antigen, which are recommended by the United States Preventive Services Task Force for screening all individuals aged 15–65 years [15]. For hepatitis C, clinicians should use an anti-HCV antibody test (e.g., enzyme immunoassay), followed by a nucleic acid test to detect and confirm the presence of HCV RNA [16]. Current syphilis tests rely on the detection of antibodies, with a traditional algorithm involving an initial nontreponemal test (e.g., Venereal Disease Research Laboratory [VDRL] or rapid plasma reagin [RPR] test) followed by a confirmatory treponemal antibody detection test (e.g., *T. pallidum* particle agglutination [TP-PA] test) [17].

HIV Self-Testing (HIVST) programs are a public health strategy designed to decentralize testing modalities and empower individuals in managing their disease, with the goal of diagnosing vulnerable populations and preventing adverse health outcomes [18]. Currently available self-testing strategies include immunoassays for the qualitative detection of HIV-1 and HIV-2 antibodies in oral fluid [19].

Given this scenario, the international scientific community proposes expanding the testing strategies more decentralized [20,21]. Technologies, such as rapid tests and self-tests, are currently the major players in improving accurate HIV screening methods, which are widely acceptable and easily accessible to increase diagnostic rates [1,9]. However, the decentralized HIV testing proposals generated controversy in the 80s, once conventional centralized tests were believed to be more efficient, with higher throughput, and easier to manage and quality assure than decentralized testing [22,23].

This debate rekindled in Brazil in January 2019 when the government started to distribute HIVST free of charge through the Brazilian National Health System (SUS) [19]. In this context, the Brazilian government acquired 400,000 tests as part of a pilot project. Following its success, this strategy was adopted as public policy, allowing the acquisition of over 600,000 tests in 2021 and 2022 [19,24]. Although the scientific literature is already contemplated by robust studies on the usability and acceptance of HIVST, the reality in countries with a high inequality level, such as Brazil, needs to be more represented [25]. This study aimed to examine the challenges associated with HIVST, such as proficiency in performing when applied to a population without any technical experience resident in Brazil.

## 2 Material and methods

**Study design.** We conducted a cross-sectional study using a convenience sample. The study occurred in sites within the metropolitan region of Belo Horizonte in the State of Minas Gerais, Southeastern Brazil, between August and November of 2019. Volunteers who obtained negative results for STI were not followed up, while those who received a diagnosis of any pathology surveyed were referred to follow-up by the SUS.

**Participants.** We included volunteers from four locations: (1) sex work facilitators in commercial sex establishments; (2) individuals residing at the Institute for Support and Orientation to Street People, which provides shelter and rehabilitation services to unhoused individuals, people with a substance use disorder, or both; (3) incarcerated people in the Public-Private Penitentiary Complex and (4) individuals receiving services at the Social Assistance Reference Centre, a public facility assisting vulnerable populations, including unhoused, low-income people, or both. We established as inclusion criteria: (1) adults aged 18 years and over; (2) literate (being able to read, write and understand Portuguese); (3) consented to perform tests for the following STI agents: HIV, hepatitis C virus (HCV) and *Syphilis.* Volunteers who met any of the following criteria were excluded: (1) Previous diagnosis of HIV infection; (2) Previous participation in a clinical trial of an HIV vaccine; (3) Previous performance of a self-test for HIV on another occasion.

**STI tests.** We used the same sample for all tests performed. The sample collection was performed using fingerstick whole blood samples drawn from each participant. These samples were used to perform the rapid tests Alere Determine HIV-1/2 (Abbott®), HCV antibody ECO test (Ecodiagnostica®), and Syphilis ECO test (Ecodiagnostica®). All tests were performed simultaneously during the same visit, following the manufacturer's instructions.

After completing previous tests, volunteers were invited to perform the HIV Detect Oral self-test (Ecodiagnostica®) by oral fluid. In this step, we evaluated the participant's ability to perform, read, and interpret the results correctly. The researcher supervised the procedure and assessed, without interference, the volunteer's task. The researcher used a structured script to classify each step as “correct” or “incorrect".

• The **Alere Determine HIV-1/2** is an in vitro, visually read, qualitative immunoassay designed for the detection of antibodies to HIV-1 and HIV-2 in human serum, plasma, or whole blood. It serves as an aid in detecting antibodies in individuals infected with HIV-1 or HIV-2. The overall sensitivity and specificity reported by the manufacturer are 100 % and 99.75 %, respectively.
• The **HCV antibody ECO test** detects antibodies to the hepatitis C virus, also using an immunochromatographic principle.
• The **Syphilis ECO test** detects antibodies to *T. pallidum* based on the same immunochromatographic principle.
• The **HIV Detect Oral Self-Test** is a qualitative immunochromatographic assay used to detect HIV-1 and HIV-2 antibodies in oral fluid.

**Questionnaire survey.** We applied a structured survey to the individuals to characterize the tested population in the following aspects: sociodemographic characteristics, sexual orientation, gender identity, sexual behavior, use of alcohol or illicit drugs, STI testing frequency, discrimination or violence, and thoughts and feelings about HIVST. The questionnaires employed are provided in the Supplementary Material.

**Data analysis**. The numerical variables were presented as mean ± standard deviation and median (interquartile distance), and categorical variables as absolute and/or relative frequencies. A logistic regression analysis was performed to evaluate the association between the level of education (as an independent variable) and test performance (correct vs. incorrect) as the dependent variable. Education level was coded numerically from 1 to 9, representing different categories of educational attainment. The results were expressed as odds ratios with 95 % confidence intervals. All analyses were conducted using R software version 4.3.2.

**Ethics statement.** These studies were performed under protocols reviewed and approved by the Ethics and Research Committee of the Faculty of Medical Sciences of Minas Gerais (CEPCM-MG protocol 12603219.5.0000.5134). Only adults aged 18 years or older were enrolled in the study, and all participants signed written informed consent to participate.

## 3 Results

### 3.1 Characterization of the study population

We included a sample of 125 participants after excluding 14 individuals who were under the influence of drugs. This diverse sample represented key population groups relevant to HIV diagnosis, with some participants belonging to more than one group. Specifically, 61 participants were sex work facilitators (48.8 %), 42 had a substance use disorder (33.6 %), 29 were incarcerated (23.2 %), and 23 self-identified as non-heterosexual (18.4 %). A summary of these population groups and their distribution is presented at the beginning of Table 1, along with detailed sociodemographic characteristics. Most volunteers identified as being of black ethnicity (80.8 %), followed a religious affiliation (76.8 %), identified as women (55.2 %), and were not in stable relationships (55.2 %). Additionally, 59.2 % reported having regular employment, while 23.2 % declared receiving an income below the Brazilian minimum wage, and 14.4 % had not completed high school.

**Table 1:** – Sociodemographic characteristics.

	Statistics
Age	33.5^a^ ± 11.1^b^ 32.0^c^ (25.0^d^–40.0^e^)
Key-population groups	
Sex-work facilitators	61 (48.8 %)
Individuals with a substance use disorder	42 (33.6 %)
Incarcerated individuals	29 (23.2 %)
Non-heterosexual individuals	23 (18.4 %)
Relationship status (n = 125)	
Single/alone	69 (55.2 %)
Married/cohabitation with a woman	12 (9.6 %)
Married/cohabitation with a man	6 (4.8 %)
Dating	19 (15.2 %)
“Hooking up”	6 (4.8 %)
Other	11 (8.8 %)
Did not answer	2 (1.6 %)
Living situation (n = 125)	
Living with parents/relatives	16 (12.8 %)
Living with wife/Kids	12 (9.6 %)
Living with a partner	6 (4.8 %)
Living with friends	1 (0.8 %)
Living lone	46 (36.8 %)
Living in an off-campus student residency	0 (0.0 %)
Other situations	14 (11.2 %)
Did not answer	1 (0.8 %)
Education Level (n = 125)	
Incomplete elementary school	36 (28.8 %)
Complete elementary school	12 (9.6 %)
Incomplete high school	18 (14.4 %)
Complete high school	46 (36.8 %)
Incomplete college education	4 (3.2 %)
Complete college education	9 (7.2 %)
Currently employed (n = 125)	
Yes	74 (59.2 %)
No	51 (40.8 %)
If not (n = 51)	
Unemployed	10 (19.6 %)
Student	1 (2.0 %)
Other	3 (5.9 %)
Does not apply	8 (15.7 %)
Prisoner	29 (56.9 %)
Current employment (n = 125)	
Public Server	1 (0.8 %)
Formally employed	5 (4.0 %)
Informally employed	1 (0.8 %)
Self-employed	8 (6.4 %)
Casual job	1 (0.8 %)
At-home partner	2 (1.6 %)
Other	8 (6.4 %)
Does not apply	40 (32.0 %)
Income of the last month (n = 125)	
Up to 1x the Brazilian minimum wage	29 (23.2 %)
From 1- 3x the Brazilian minimum wage	15 (12.0 %)
From 3 - 6x the Brazilian minimum wage	23 (18.4 %)
From 6 - 9x the Brazilian minimum wage	14 (11.2 %)
From 9 - 12x the Brazilian minimum wage	11 (8.8 %)
From 12 - 15x the Brazilian minimum wage	1 (0.8 %)
More than 15x the Brazilian minimum wage	1 (0.8 %)
Did not answer	1 (0.8 %)
Does not know	1 (0.8 %)
Does not have income	29 (23.2 %)
Monthly family income (n = 125)	
Does not have income	33 (26.4 %)
Up to 1x the Brazilian minimum wage	25 (20.0 %)
From 1 - 3x the Brazilian minimum wage	14 (11.2 %)
From 3 - 6x Brazilian minimum wage	19 (15.2 %)
From 6 - 9x the Brazilian minimum wage	14 (11.2 %)
From 9 - 12x the Brazilian minimum wage	14 (11.2 %)
From 12 - 15x the Brazilian minimum wage	3 (2.4 %)
More than 15 x the Brazilian minimum wage	2 (1.6 %)
Does not know	1 (0.8 %)
Number of people depending on the family income (n = 125)	
One	37 (29.6 %)
Two	18 (14.4 %)
Three	19 (15.2 %)
Four	8 (6.4 %)
Five	11 (8.8 %)
Six	2 (1.6 %)
Other	4 (3.2 %)
Does not have income	26 (20.8 %)
Religion (n = 125)	
None	29 (23.2 %)
Christian	30 (24.0 %)
Spiritism	16 (12.8 %)
Afro-brazilian (umbanda/candomblé)	9 (7.2 %)
Protestant	31 (24.8 %)
Other	10 (8.0 %)
Self-declared ethnicity (n = 125)	
White	20 (16.0 %)
Asian	3 (2.4 %)
Black	101 (80.8 %)
ndigenous	1 (0.8 %)
Sexual orientation (n = 125)	
Heterossexual	102 (81.6 %)
Homossexual	3 (2.4 %)
Bissexual	20 (16.0 %)
Gender identity (n = 125)	
Cis Man	56 (44.8 %)
Cis Woman	69 (55.2 %)

n Sample size.

a Mean.

b Standard deviation.

c Median.

d First e Third quartile.

### 3.2 Awareness of HIV testing and HIVST

Despite a significant number of participants having undergone HIV testing, the awareness of HIVST was limited. An evaluation of the participant's knowledge about STI testing revealed that most had been tested for STIs (64.5 %), with the majority seeking testing due to concerns about HIV contamination (95.2 %). While 66.9 % of volunteers knew that the Brazilian Unified Health System (SUS) offers free testing services, a substantial proportion (73.8 %) were unaware of the existence of self-testing technology (Table 2).

**Table 2:** – Testing habits

	Statistics
Previous testing for STIs^a^ (n = 124)	
Yes, in the private network of diagnostic medicine laboratories	25 (20.2 %)
Yes, in the public health system through a rapid test	56 (45.2 %)
Yes, through a self-test purchased at a pharmacy	2 (1.6 %)
Yes, through a self-test provided by SUS^b^	6 (4.8 %)
I have never tested for STIs	44 (35.5 %)
Main motivation for seeking STI testing (n = 124)	
To know my HIV contamination status	118(95.2 %)
To know my Hepatitis B and C contamination status	1 (0.8 %)
To know my Syphilis contamination status	2 (1.6 %)
To know other diseases' contamination status	3 (2.4 %)

Main motivation for seeking HIV testing? (n = 118)	
I do not know my partner's contamination status	15 (12.7 %)
The condom broke or slipped during sex	12 (10.2 %)
I believe I have had high-risk sexual behavior	45 (38.1 %)
I do periodic tests regardless of personal risk	15 (12.7 %)
Being a prisoner	33 (28.0 %)
Previous knowledge of SUS performing HIV and other STIs tests free of charge through testing and counseling centers (CTAs), in some health centers and in emergency care units (n = 124)	
I did not know	41 (33.1 %)
Yes, I knew and I have already visited these institutions to get tested	39 (31.5 %)
Yes, I knew, but I never visited any of these institutions to test myself for STIs	44 (35.5 %)
Previous knowledge of the HIV self-test by oral fluid? (n = 122)	
Yes	32 (26.2 %)
No	90 (73.8 %)
Previous knowledge of HIV self-test by oral fluid being sold at pharmacies? (n = 122)	
Yes	18 (14.8 %)
No	104 (85.2 %)

n Sample size.

*Note*: Participants could choose more than one testing modality, resulting in percentages that exceed 100 %.

a Sexually Transmitted Infection.

b Brazilian National Health System.

## 4 Performance of HIVST and pre-analytical errors

After assessing participants' prior knowledge of HIVST, they were invited to perform the test. During this process, the ability of volunteers to execute the self-test on their own was evaluated (Table 3). The results indicated that most interviewees (93.5 %) encountered difficulties performing at least one step outlined in the test manual. Notably, 61 % of individuals committed at least one significant deviation, with the most common errors including failure to perform oral cleaning before the test (74.2 %), neglecting to make the prescribed up-and-down movements with the swab inside the tube (56.8 %), and forgetting to set the timer for result evaluation (56.1 %).

**Table 3:** Researcher's evaluation OF HIV self-test steps performed by participants.

	Performed correctly	Performed incorrectly
Performed mouth cleaning (n = 124)	32 (25.8 %)	92 (74.2 %)
Inverted the tube with the diluent solution to homogenize (n = 124)	66 (53.2 %)	58 (46.8 %)
Place the tube with the diluent solution in the indicated location on the result card (n = 124)	83 (66.9 %)	41 (33.1 %)
Opened the package containing the Swab for sample collection without touching the collection area with their fingers (n = 124)^a^	99 (79.8 %)	25 (20.2 %)
Gently swabbed the upper gum several times, going from one corner to another (n = 124)	73 (58.9 %)	51 (41.1 %)
Placed the swab for sample collection in the tube with the diluent solution and made light movements up and down, touching the swab against the wall of the tube so that the sample is mixed in the diluent solution. When removing the Swab, pressed on the tube wall to leave all the liquid contained in the swab inside the tube. (n = 124)	53 (42.7 %)	71 (57.3 %)
If the tube with the diluent solution has been removed from the location indicated on the result card for this procedure, the tube must return to the location indicated later. (n = 124)	93 (75 %)	31 (25 %)
Discarded the test if the liquid got very cloudy. (n = 124)	93 (75 %)	31 (25 %)
Removed the test strip from the aluminum package and did not touch the area below the arrows (MAX) (n = 124)	74 (59.7 %)	50 (40.3 %)
Inserted the test strip into the tube containing the mixed sample without the sample level exceeding the MAX line (n = 124)	100 (80.6 %)	24 (19.4 %)
Started counting the time after inserting the test strip in the tube (n = 123)	54 (43.9 %)	69 (56.1 %)
Left the test strip for 20 min inside the tube containing the sample mixed with the diluent solution (n = 122)^a^	73 (59.8 %)	49 (40.2 %)
Read the result between 20 and 30 min (n = 121)^a^	73 (60.3 %)	48 (39.7 %)

		Statistics

Did the volunteer ask the research team questions? (n = 114)		
No		23 (20.2 %)
Yes		90 (78.9 %)
Invalid		1 (0.9 %)
At what stage? (n = 90)		
There was no doubt		7 (7.8 %)
At the beginning		35 (38.9 %)
During the sample collection		55 (61.1 %)
During insertion		21 (23.3 %)
During the test strip reading		25 (27.8 %)

n Sample size.

a Serious errors according to the test manufacturer.

### 4.1 User experience and willingness to pay for HIVST

Following the self-tests, participants provided feedback on their user experience. An overwhelming majority (95 %) found the test steps “easy or very easy” to perform. Most participants (97.4 %) reported reading the results as “well or very well,” which boosted their confidence in the outcome. Interestingly, participants who had not previously used this testing modality expressed increased motivation for frequent self-testing (74.2 %). However, considering the average selling price in Brazil, 70.58 % of participants indicated they would not be willing to purchase the test (Table S4). Importantly, no coercion was reported during the testing, and all volunteers stated that they had voluntarily chosen to undergo testing.

### 4.2 Comparison of HIVST results with gold standard test

The results of the HIV self-test (HIVST) were compared to those of the gold standard HIV test. From the health professional's perspective, the HIVST demonstrated 100 % agreement, achieving 100 % sensitivity and 100 % specificity in comparison to the gold standard. The prevalence of HIV in our sample was 1.6 %, with two individuals testing positive on both the Alere Determine HIV-1/2 test (Abbott®) and the HIV Detect Oral self-test (Ecodiagnostica®). However, from the patient's perspective, the HIVST had a sensitivity of 100 % and a specificity of 95.1 %, with 5 % of the results being misinterpreted (Table 4).

**Table 4:** Volunteers’ accuracy in interpreting HIV self-test results.

	Negative	Positive	Invalid
Result reading according to the volunteer (n = 120)	110 (91.7 %)	8 (6.7 %)	2 (1.7 %)
Result reading according to the observer (n = 120)	116 (96.7 %)	2 (1.7 %)	2 (1.7 %)

	*No*	*Yes*	*Invalid*

Was the volunteer's reading correct? (n = 120)	6 (5 %)	114 (95 %)	2 (1.7 %)

Sensitivity/Specificity		100 %/95.1 %	

n Sample size.

### 4.3 Potential impact on the detection of other STIs

In addition to HIV testing, samples were screened for HCV and syphilis. The results revealed a significant number of cases of syphilis (19.2 %) and HCV (4.8 %). If only HIV testing had been performed, at least 22.4 % of individuals with other STIs would have remained unaware of their infection status.

### 4.4 Understanding of the window period

Concerns were raised regarding participants' understanding of the appropriate timing for HIVST after a risky situation. When asked initially, only a tiny minority (4.8 %) provided the correct answer (Table 5). Even after reading the test instructions, only one participant modified their response to the question, emphasizing the need for further education and awareness.

**Table 5:** HOW long after a risky situation (having sex without a condom and sharing needles) should YOU perform a rapid HIV test or self-test?

	Before (n = 124)	After (n = 119)
Soon after a risky situation	42 (33.9 %)	48 (40.3 %)
24 h after a risky situation	33 (26.6 %)	34 (28.6 %)
15 days after a risky situation	7 (5.6 %)	8 (6.7 %)
30 days after a risky situation	6 (4.8 %)	7 (5.9 %)
3 months after a risky situation	10 (8.1 %)	7 (5.9 %)
I do not know	23 (18.5 %)	15 (12.6 %)
Did not answer	3 (2.4 %)	0 (0.0 %)

n Sample size.

### 4.5 Correlation between education level and HIVST performance

The logistic regression analysis identified an odds ratio (OR) of 0.95 for education level (95 % CI: 0.54–1.61; p = 0.85), suggesting that there was no significant association between education level and the likelihood of correctly performing the HIVST. Additionally, the intercept had an OR of 0.06 (95 % CI: 0.008–0.28; p = 0.001), indicating a very low baseline probability of correct test performance when education level is minimal.

## 5 Discussion

Developing countries, such as Brazil, are often underrepresented in international literature concerning the applicability of self-tests in public health, especially among populations vulnerable to sexually transmitted infections (STIs) [20,26]. This study aimed to shed light on the challenges of implementing HIVST in groups at risk for STIs. Our main findings indicate that HIVST appears well-received among key population groups. Despite high rates of procedural errors and doubts during the tests, these tests were safe, accurate, and empowered users to take control of their health. However, the high cost of HIVST significantly limits accessibility for vulnerable populations. The widespread adoption of this technology may inadvertently lead to the underdiagnosis of other STIs and potentially promote unprotected sexual intercourse among serodiscordant partners due to a misunderstanding of the immunological window concept [[27], [28], [29]].

In terms of which populations would most benefit from HIVST, it is essential to evaluate its impact on key population groups at risk of HIV infection, as the World Health Organization (WHO) emphasizes the need to prioritize their specific needs for effective health policies related to HIV [10]. Our study included the primary population groups associated with high risk for STIs. However, recruiting transgender individuals proved challenging despite visits to locations where this population is known to frequent. Additionally, some participants were sober but still exhibited residual effects of drug use during the interviews, which may have influenced the accuracy of their responses.

One of the main controversies surrounding HIVST in a developing country context is the potential for coercion, particularly in environments such as brothels, detention centers, and high-social vulnerability settings [30]. While studies have reported instances of coercion in specific settings [24,29,31], our study did not identify such situations. All volunteers reported testing of their own free will. Furthermore, our study highlighted an aspect less commonly discussed in international literature: the prevalence of pre-analytical and reading errors. The high rates of procedural errors (93.5 %) and doubts about test performance (78.9 %) observed in our study are closely linked to the vulnerability of the sample in a developing country context. Most participants had not completed high school, and many were unemployed or earning less than the Brazilian minimum wage. While these findings are concerning, they reflect the broader socio-economic challenges faced by many in Brazil [32,33]. Importantly, pre-analytical errors did not compromise the accuracy of results, as HIVST demonstrated 100 % agreement with the gold standard test, even though a 5 % error rate occurred when volunteers interpreted the test results themselves.

Despite the high rates of procedural errors, our study reinforces that self-tests empower users to take control of their health. Previous studies in different settings have shown that individuals find self-tests easy to use and interpret [26,27,[29], [30], [31], [32], [33]]. In our study, participants who had never used this testing modality before reported increased motivation to test themselves regularly after their experience with HIVST. Additionally, the majority expressed confidence in their ability to read test results accurately.

The ability to correctly read the test results was hypothesized to improve with higher levels of education. However, our logistic regression analysis did not identify a statistically significant relationship between the ability to read correctly and the level of education, as indicated by the non-significant coefficient and OR. This outcome may be attributed to the sample size limitations and the low frequency of incorrectly reading the results. Nevertheless, the intercept suggests a very low baseline probability of correctly interpreting test results when the level of education is minimal, highlighting the potential impact of lower educational attainment on test performance.

However, while self-tests offer numerous benefits, their high cost poses a significant barrier to accessibility for key populations that would benefit most from HIVST. Our study aligns with research in other developing countries, which has demonstrated that many individuals may be unwilling to pay the market price for HIVST. This limitation becomes particularly relevant when considering that HIVST distribution programs may inadvertently reduce diagnosis rates for other STIs and potentially promote unprotected sexual intercourse among those infected with other STIs.

Our study revealed that most participants sought STI testing primarily due to concerns about HIV contamination (95.2 %). Notably, a considerable number of participants also tested positive for Syphilis (19.2 %) and Hepatitis C (4.8 %). This suggests that solely relying on HIV testing may lead to the underdiagnosis of other STIs and potentially enable unprotected sexual intercourse among serodiscordant partners. In 2019, the Brazilian government initiated a pilot project approving the distribution of HIVST to key populations, encouraging non-mandatory testing for other STIs. However, this approach contrasts with a 28.3 % increase in new Syphilis cases in 2019 compared to the previous year [34]. Although our study did not directly address these issues, our findings underscore the need for further research on the potential impact of increased HIVST availability on rates of other STIs and the importance of targeted interventions and counseling in distribution programs.

Another critical concern related to underdiagnosis is the misunderstanding of the concept of the window period, potentially leading to a false sense of security. Despite explanatory materials included in HIVST kits about the window period and signs of acute retroviral syndrome, our results indicate that only a small fraction of participants (4.8 %) understood the correct definition of the HIV window period. Even after reading the provided materials, only one participant's understanding improved. These findings emphasize the importance of public health initiatives that combine HIVST distribution with education, particularly among key populations in developing countries.

One of this study's key limitations is the sample composition. While efforts were made to include key population groups at risk for STIs, recruiting transgender individuals proved challenging. This limitation may have implications for the generalizability of the findings to the broader population of key populations. While HIVST was generally perceived as empowering, there is a need to ensure that users understand its limitations and the importance of seeking medical advice when necessary. Misinterpretation of empowerment may lead to an overreliance on self-testing and delayed healthcare-seeking behaviors [35]. Additionally, the study was conducted in a specific geographic location (Belo Horizonte, Brazil), and findings may not directly apply to other regions with different sociodemographic characteristics.

In conclusion, our study supports the notion that HIVST seems to be accurate, safe, and able to empower users to take an active role in managing their health. However, the high cost of HIVST limits its accessibility for vulnerable populations, and its widespread use may inadvertently reduce diagnosis rates of other STIs and promote unprotected sexual intercourse due to misconceptions about the window period. Further research is needed to understand the long-term impact of HIVST use, particularly about increasing rates of STIs and addressing gaps in education, especially among key populations in developing countries.

## Author statements

This work was supported by the 10.13039/501100010301Faculty of Medical Sciences of Minas Gerais, Belo Horizonte, Minas Gerais, Brazil. These studies were performed under protocols reviewed and approved by the Ethics and Research Committee of the Faculty of Medical Sciences of Minas Gerais (CEPCM-MG protocol 12603219.5.0000.5134). Only adults aged 18 years or older were enrolled in the study, and all participants signed written informed consent to participate. The authors have no competing interests to declare.

## Author's statement

João Vitor Innecco Arêas: Conceptualization, Methodology, Validation, Formal analysis, Investigation, Writing - Original Draft, Funding acquisition. Gabriela Bragança Costa e Moreira: Investigation, Writing – Original Draft. Gabriela Baêta Picchioni: Investigation, Writing – Original Draft. João Vitor Levindo Coelho Novaes: Methodology, Formal analysis, Writing - Review & Editing, Visualization. Luísa Castro de Sousa Pires: Investigation, Writing – Original Draft. Luara Isabela dos Santos: Conceptualization, Methodology, Supervision, Writing, Project administration.

## Declaration of competing interest

The authors declare that they have no known competing financial interests or personal relationships that could have appeared to influence the work reported in this paper.
