# Supplementary material for: Assessing the impact of HIV self-testing on diagnosis rates in vulnerable groups in belo horizonte, Brazil: A cross-sectional analysis
Source: Public Health Pract (Oxf). 2024 Dec 22;9:100567. doi: 10.1016/j.puhip.2024.100567 (PMC11742301; doi:10.1016/j.puhip.2024.100567)
Supplement: Multimedia component 1 [file mmc1.docx]

**SUPPLEMENTARY MATERIAL**

**For**

**Assessing the Impact of HIV Self-Testing on Diagnosis Rates in Vulnerable Groups in Belo Horizonte, Brazil: A Cross-Sectional Analysis**

**Arêas et al., 2024**

**TABLE OF CONTENTS**

**Table S1.** SCREENING CRITERIA AND ELIGIBILITY ASSESSMENTS QUESTIONNAIRE **2**

**Table S2.** PARTICIPANT PROFILE QUESTIONNAIRE  **3**

**Table S3**. HIV DETECT ORAL SELF-TEST SATISFACTION QUESTIONNAIRE (*ECODIAGNÓSTICA*™) **29**

**Table S2.** SCREENING CRITERIA AND ELIGIBILITY ASSESSMENTS QUESTIONNAIRE

Volunteer Code: ______ Initials: ___________
Researcher Code: ______
Date: **/**/_____ Start Time: _________

**Inclusion Criteria for the Volunteer**
Selection criteria 1, 2, 3, 4, 5, 6, and 7 must be satisfied for INCLUSION in the study. If criterion 3 is marked as "No," the patient should be considered "Temporarily Ineligible."

1. Is the participant ≥ 18 years old?
   ( ) Yes ( ) No
2. Does the participant have the ability to understand Portuguese?
   ( ) Yes ( ) No
3. Does the participant deny engaging in unprotected sex or needle sharing in the last 30 days?
   ( ) Yes ( ) No
4. Does the participant consent to testing for all the following STIs: HIV, HCV, and Syphilis?
   ( ) Yes ( ) No
5. Does the participant deny having prior technical experience with blood or oral fluid collection?
   ( ) Yes ( ) No
6. Does the participant deny having been previously diagnosed with HIV?
   ( ) Yes ( ) No
7. Does the participant deny participating in any HIV vaccine clinical trial?
   ( ) Yes ( ) No

**After the evaluation, the volunteer was considered:**
( ) Eligible ( ) Ineligible ( ) Temporarily Ineligible

**Observations:**

**Professional’s Signature: _______________________________**

**NOTE**: Remind the volunteer that they will now collect material for blood and oral fluid tests.

**Table S3.** PARTICIPANT PROFILE QUESTIONNAIRE

Volunteer Code: ______ Initials: ___________ Researcher Code:______

Date: ____/____/_____ Start Time: _________

1. How long after a risk exposure (having unprotected sex and sharing needles) should you take a rapid HIV test or self-test?

( ) Immediately after the risky behavior

( ) 24 hours after the risky behavior

( ) 15 days after the risky behavior

( ) 30 days after the risky behavior

( ) 3 months after the risky behavior

( ) I don't know

( ) Did not answer

**SECTION – SOCIODEMOGRAPHIC CHARACTERISTICS**

2. How old are you? ___________

3. Date of birth: ___/___/____

4. Currently, you are:

( ) Single/Alone

( ) Married to a woman / In a stable union with a woman

( ) Married to a man / In a stable union with a man

( ) Dating

( ) Casually seeing someone

( ) Other. Specify: __________________

( ) Did not answer

5. Who do you currently live with?

( ) Parents/relatives

( ) Woman/children

( ) Partner

( ) Friend

( ) Alone

( ) College roommates

( ) Other situations. Specify: _________

( ) Did not answer

6. Education level:

( ) Incomplete primary education

( ) Completed primary education

( ) Incomplete high school

( ) Completed high school

( ) Incomplete higher education

( ) Completed higher education

( ) No formal education

( ) Did not answer

( ) Does not know

7. Are you currently working?

( ) Yes (skip to question 7.2)

( ) No (skip to question 7.1)

( ) Did not answer

7.1 You are: (For those not working)

( ) Never worked

( ) Unemployed

( ) Student

( ) On medical leave for ___ years and/or __ months

( ) Retired due to length of service

( ) Retired due to permanent disability

( ) Other, specify: __________________

( ) Not applicable

( ) Did not answer

7.2 You are:

( ) Public servant

( ) Employee with a formal work contract

( ) Employee without a formal work contract

( ) Self-employed

( ) Occasional jobs

( ) Intern

( ) Domestic worker

( ) Housewife

( ) Sex worker

( ) Employer/business owner

( ) Other_________________

( ) Not applicable

( ) Did not answer

8. What was your income last month? R$: _________________

( ) Up to 1 minimum wage (up to R$ 998.00)

( ) From 1 to 3 minimum wages (R$ 998.01 to R$ 2,994.00)

( ) From 3 to 6 minimum wages (R$ 2,994.01 to R$ 5,988.00)

( ) From 6 to 9 minimum wages (R$ 5,988.01 to R$ 8,982.00)

( ) From 9 to 12 minimum wages (R$ 8,982.01 to R$ 11,976.00)

( ) From 12 to 15 minimum wages (R$ 11,976.01 to R$ 14,970.00)

( ) More than 15 minimum wages (more than R$ 14,970.01)

( ) Did not answer

( ) Does not know

( ) No income

9. Adding your income to the income of the people who live with you, what is the approximate monthly household income?

( ) No income

( ) Up to 1 minimum wage (up to R$ 998.00)

( ) From 1 to 3 minimum wages (R$ 998.01 to R$ 2,994.00)

( ) From 3 to 6 minimum wages (R$ 2,994.01 to R$ 5,988.00)

( ) From 6 to 9 minimum wages (R$ 5,988.01 to R$ 8,982.00)

( ) From 9 to 12 minimum wages (R$ 8,982.01 to R$ 11,976.00)

( ) From 12 to 15 minimum wages (R$ 11,976.01 to R$ 14,970.00)

( ) More than 15 minimum wages (more than R$ 14,970.01)

( ) Did not answer

( ) Does not know

10. How many people live off the household's monthly income? (including yourself)

( ) One

( ) Two

( ) Three

( ) Four

( ) Five

( ) Six

( ) Other: ____

11. Do you practice any religion or worship?

( ) No

( ) Catholic

( ) Spiritist

( ) Afro-Brazilian (Umbanda/Candomblé)

( ) Jewish

( ) Evangelical/Protestant

( ) Oriental/Buddhism

( ) Other. Which?__________________

( ) Did not answer

( ) Does not know

12. We would now like to know your race/ethnicity (Spontaneous response):

( ) White

( ) Yellow

( ) Mixed-race

( ) Black

( ) Indigenous

( ) Did not answer

( ) Does not know

**SECTION – SEXUAL ORIENTATION, GENDER IDENTITY, PLACES TO MEET PARTNERS**

Now, we would like to know about your sexual orientation and gender identity – meaning: who you have sex with and your feelings about it.

13. Regarding your sexual orientation, you are:

( ) Heterosexual

( ) Homosexual

( ) Bisexual

( ) Queer

( ) Pansexual

( ) Intersex

( ) Other: _________

( ) Did not answer

14. Regarding your gender identity, you are:

( ) Cis man

( ) Cis woman

( ) Trans woman

( ) Trans man

( ) Travesti

( ) Other: _________

( ) Did not answer

15. Regarding sexual behavior, in the past 6 months, you had sex:

( ) Only with men

( ) Only with women

( ) Primarily with men, but occasionally with women

( ) Primarily with women, but occasionally with men

( ) Equally with men and women

( ) Did not have sex

( ) No response

16. Regarding sexual attraction, in the past 6 months, you:

( ) Felt attraction only to men

( ) Felt attraction only to women

( ) Felt primarily attracted to men, but occasionally to women

( ) Felt primarily attracted to women, but occasionally to men

( ) Felt equally attracted to men and women

( ) Did not feel sexual attraction

( ) No response

17. In the past 6 months, did you visit any places or use any devices to find, meet, or interact with sexual partners?

( ) Yes

( ) No – Skip to question 19

( ) Don't remember/not sure – Skip to question 19

( ) No response

18. What places did you visit or what devices did you use to find, meet, or interact with sexual partners?

( ) Nightclub

( ) Bars

( ) Sauna

( ) Cinema / Erotic booths

( ) Hook-up spots. Which one? ________________________

( ) Streets/squares/parks

( ) Work/School/University/Church

( ) Friends' house

( ) Internet

( ) Mobile app. Which one? ( ) Grindr ( ) Scruff ( ) Hornet ( ) Tinder ( ) Growlr ( ) Other

( ) Other ________________________

( ) Not applicable

( ) No response

**SECTION – STIs, OTHER DISEASES, AND RISK PERCEPTION**

19. In the past 6 months, have you experienced any of the following symptoms? (You may select more than one option)

( ) Urethral or rectal discharge with a different color or bad smell

( ) Ulcers or sores on the penis or anus

( ) Warts on the penis or anus

( ) Small blisters on the penis or anus

( ) Ulcers or sores on the vagina

( ) Warts on the vagina or anus

( ) Small blisters on the vagina or anus

( ) Molluscum contagiosum (hand-foot-mouth)

( ) I was diagnosed with an STI (e.g., gonorrhea, syphilis, herpes, chancroid, pubic lice, genital warts). __________ times, in the past 6 months

( ) Other: ________________________

( ) Did not have any of the mentioned problems (Skip to question 21)

( ) Don't remember/Not sure

( ) No response

20. Did you receive treatment for any of these STIs?

( ) Yes

( ) No

( ) Self-medicated

( ) Not applicable

( ) No response

( ) Not sure

21. Do you think you are at risk of contracting HIV? (First mark “NO” or “YES”, then read the alternatives. You can select more than one.)

( ) No

( ) I always have protected sex

( ) I trust my prevention methods

( ) I have a reliable steady partner

( ) I don’t have many partners/I am not promiscuous

( ) I carefully choose my partners

( ) I have a steady partner with a recent negative test result

( ) I had brief contact (rubbing)

( ) My partner and I are both HIV-negative and regularly test

( ) Other: ________________________

( ) Don’t know

( ) Yes

( ) The condom might break or tear

( ) I prefer sex without a condom

( ) I am homosexual/bisexual

( ) I have an HIV-positive partner

( ) I have difficulty controlling sexual impulses

( ) I sometimes have unprotected sex (oral, anal, vaginal)

( ) I have many sexual partners

( ) I visit saunas or hook-up locations

( ) I drank/used drugs and didn’t take precautions

( ) I had brief contact (rubbing)

( ) Other: ________________________

( ) Don’t know

( ) No response

22. Have you been diagnosed with any of the following conditions by a doctor?

( ) Respiratory disease (COPD, asthma, bronchitis)

( ) Depression

( ) Anxiety

( ) Bipolar disorder

( ) Cancer (specify) ________________________

( ) Autoimmune disease (specify) ________________________

( ) Other: ________________________

**SECTION – TYPES OF PARTNERS, SEXUAL PRACTICES, AND CONDOM USE**

Please respond specifically regarding your sexual relations with men and/or women.

We define: (READ THE DEFINITIONS)

FIXED PARTNERS: those with whom you had sexual relations within the context of a planned meeting, a relationship, a casual relationship, or any emotional involvement.

OCCASIONAL PARTNERS: those with whom you had sexual relations one or more times without planning the next meeting, including unknown partners.

23. Have you had the desire to get pregnant or have more children in the six months prior to the interview (question for heterosexual men)?

( ) Yes, I wanted to impregnate my partner in the past six months.

( ) No

24. Did your partner want to get pregnant in the past six months?

( ) Yes

( ) No

**FIXED PARTNERS (MEN)**

25. Did you have fixed male partners in the past 6 months?

( ) Yes. How many? __________

( ) No. Skip to question 29, regarding occasional partners

( ) No response

26. Did you have receptive anal sex (were penetrated) with fixed partner(s) in the past 6 months?

( ) No. Skip to question 27, regarding insertive anal sex

( ) Yes. How often did you use a condom?

( ) Always

( ) Most of the time

( ) Sometimes

( ) Rarely

( ) Did not use a condom in the past 6 months

( ) Don’t remember

( ) Not applicable (did not have FP)

( ) No response

27. Did you have insertive anal sex (penetrated) with fixed partner(s) in the past 6 months?

( ) No

( ) Yes. How often did you use a condom?

( ) Always

( ) Most of the time

( ) Sometimes

( ) Rarely

( ) Did not use a condom in the past 6 months

( ) Don’t remember

( ) Not applicable (did not have FP)

( ) No response

28. Did any of these fixed partner(s) have a positive HIV test result?

( ) Yes; How many? _______

( ) No; How many? _______

( ) Don’t know, unsure of the test result, how many? _______

( ) Not applicable

( ) No response

**OCCASIONAL PARTNERS (MEN)**

29. Did you have occasional male partners in the past 6 months?

( ) Yes. How many? _______

( ) No. Skip to question 33, regarding fixed female partners.

( ) No response

29.1 How many of these were unknown? __________

30. Did you have receptive anal sex (were penetrated) with occasional partner(s) in the past 6 months?

( ) No. Skip to question 31, regarding insertive anal sex.

( ) Yes. How often did you use a condom?

( ) Always

( ) Most of the time

( ) Sometimes

( ) Rarely

( ) Did not use a condom in the past 6 months

( ) Don’t remember

( ) Not applicable (did not have OP)

( ) No response

31. Did you have insertive anal sex (penetrated) with occasional partner(s) in the past 6 months?

( ) No

( ) Yes. How often did you use a condom?

( ) Always

( ) Most of the time

( ) Sometimes

( ) Rarely

( ) Did not use a condom in the past 6 months

( ) Don’t remember

( ) Not applicable (did not have OP)

( ) No response

32. Did any of these occasional partner(s) have a positive HIV test result?

( ) Yes; How many? _______

( ) No; How many? _______

( ) Don’t know, unsure of the test result, how many? _______

( ) Not applicable

( ) No response

**For those who had sexual relations with women, otherwise skip to question 35**

About your sexual relations with women:

**FIXED PARTNERS (WOMEN)**

33. Did you have fixed female partners in the past 6 months?

( ) Yes; How many? ________

( ) No. Skip to question 37, regarding occasional female partners.

( ) Never had female partners.

( ) No response

34. Did you have vaginal sex with fixed partner(s) in the past 6 months?

( ) No. Skip to question 35, regarding anal sex with fixed partner(s).

( ) Yes. How often did you use a condom?

( ) Always

( ) Most of the time

( ) Sometimes

( ) Rarely

( ) Did not use a condom in the past 6 months

( ) Don’t remember

( ) Not applicable (did not have FP/never had sexual relations with women)

( ) No response

35. In the last 6 months, have you had anal sex with regular female partner(s)?

( ) No

( ) Yes. How often did you use a condom?

( ) Always

( ) Most of the time

( ) Sometimes

( ) Rarely

( ) Did not use a condom in the last 6 months

( ) Don’t remember

( ) Not applicable (No regular female partner(s) / Never had sex with women)

( ) Did not respond

36. Did any of these regular female partners have a positive HIV test result?

( ) Yes; How many? _______

( ) No; How many? _______

( ) Don’t know, unknown test result, how many? ______

( ) Not applicable

( ) Did not respond

**OCCASIONAL FEMALE PARTNERS**

37. Have you had occasional female partners in the last 6 months?

( ) Yes. How many? _________

( ) No. Skip to question 41

( ) Never had female partners.

( ) Did not respond

37.1 Of these, how many were strangers? __________

38. In the last 6 months, have you had vaginal sex with occasional female partner(s)?

( ) No. Skip to question 39 about anal sex with occasional partner(s)

( ) Yes. How often did you use a condom?

( ) Always

( ) Most of the time

( ) Sometimes

( ) Rarely

( ) Did not use a condom in the last 6 months

( ) Don’t remember

( ) Not applicable (No occasional female partners / Never had sex with women)

( ) Did not respond

39. In the last 6 months, have you had anal sex with occasional female partner(s)?

( ) No

( ) Yes. How often did you use a condom?

( ) Always

( ) Most of the time

( ) Sometimes

( ) Rarely

( ) Did not use a condom in the last 6 months

( ) Don’t remember

( ) Not applicable (No occasional female partners / Never had sex with women)

( ) Did not respond

40. Did any of these occasional partners have a positive HIV test result?

( ) Yes; How many? _______

( ) No; How many? _______

( ) Don’t know, unknown test result, how many? ______

( ) Not applicable

( ) Did not respond

The following questions refer to sexual relations with both men and women.

41. In the last 6 months, has the condom broken or slipped off during intercourse?

( ) Yes. How many times? ________

( ) No

( ) Not applicable (Did not use condoms in the last 6 months)

42. Think about the last time you didn’t use a condom in the past 6 months. What were the reasons for not using one?

( ) Did not have a condom available

( ) Desire/passion/emotional involvement

( ) Regular/trusted partner

( ) Use of alcohol and/or drugs

( ) Did not intend for penetration to occur/quick encounter

( ) Partner requested it

( ) It was oral sex

( ) My partner is HIV+ with undetectable viral load

( ) Other: ____________________

( ) Not applicable (Always used a condom)

( ) Did not respond

( ) Don’t know

43. Thinking of the times when you did not use a condom in the last 6 months, what did you do to prevent or reduce the risk of HIV infection?

(Multiple answers possible)

( ) Did not engage in receptive sex

( ) Only performed insertive sex

( ) Only engaged in oral sex

( ) Only masturbation

( ) Played (fingering and sexual toys)

( ) Did not let the partner ejaculate in the anus

( ) Did not engage in vaginal sex

( ) Used PEP (post-exposure prophylaxis)

( ) Got regular HIV tests

( ) Asked the partner for an HIV test before engaging in sex

( ) Reduced the number of occasional partners

( ) Did not think about it, just did not use a condom

( ) Not applicable (Used condoms every time)

( ) Other: _____________________

( ) Did not respond

( ) Don’t know

Now I will ask about your sexual relations in the last 6 months, where there was payment or another exchange of favors.

44. In the last 6 months, have you accepted money, drugs, favors, valuable objects, or services in exchange for sex?

( ) Yes

( ) No. Skip to question 45

44.1 If YES, how often?

( ) Always

( ) Most of the time

( ) Sometimes

( ) Rarely

( ) Did not use a condom in the last 6 months

( ) Don’t remember

( ) Not applicable (No regular female partners / Never had sex with women)

( ) Did not respond

45. In the last 6 months, have you worked as a sex worker?

( ) Yes

( ) No

( ) Not applicable

45.1 How many commercial partners did you have sexual relations with in the last 6 months?

( ) One partner

( ) 2 to 5 partners

( ) 6 to 10 partners

( ) More than 10 partners

( ) Not applicable

( ) Did not respond

( ) Don’t know

45.2 How often did you use a condom with these partners in the last 6 months?

( ) Always

( ) Most of the time

( ) Sometimes

( ) Rarely

( ) Did not use a condom in the last 6 months

( ) Not applicable

( ) Did not respond

( ) Don’t know

**BLOCK – ALCOHOL/ILLICIT DRUG USE**

Now let's talk about alcohol and drug use in sexual activity situations.

46. In the last 6 months, did you use alcoholic beverages in flirting or sexual situations?

( ) Yes

( ) No - Skip to question 49 about illicit drug use

47. In the last 6 months, how often did you consume five or more drinks on a single occasion? (One drink is equivalent to a can of beer/cup of draft beer, or a glass of wine, or a shot (30 ml) of liquor (vodka, whisky, cachaça, liqueur, tequila, etc.).

This question refers to flirting or sexual situations.

( ) Never (consumed less than 5 drinks)

( ) Rarely

( ) Once a month

( ) Once a week

( ) Almost every day

( ) Not applicable (Did not drink)

( ) Did not respond

48. Did alcohol interfere with your use of condoms in the last 6 months?

( ) Did not interfere

( ) Made it more difficult

( ) Did not use a condom because I consumed alcohol

( ) Not applicable (Did not drink)

( ) Did not respond

( ) Don’t know

49. In the last 6 months, did you use illicit drugs in flirting or sexual situations?

( ) Yes

( ) No

50. What drugs did you use?

( ) Poppers

( ) Cocaine

( ) Crack

( ) Marijuana

( ) Club drugs (ketamine, ecstasy, LSD, GHB, bath salts, etc.)

( ) Inhalants (whippets, loló, nitrates, chloroform, or ether)

( ) Erectile stimulants (Sildenafil, Viagra®, Cialis®, Levitra®, Helleva®)

( ) Injectable drugs

( ) Not applicable

( ) Did not respond

( ) Don’t know

51. Did drug use interfere with your use of condoms in the last 6 months?

( ) Did not interfere

( ) Made it more difficult

( ) Did not use a condom because I consumed drugs

( ) Not applicable (Did not use drugs)

( ) Did not respond

( ) Don’t know

52. Have you ever used injectable drugs?

( ) No

( ) Yes, only once

( ) Yes, less than once a month

( ) Yes, at least once a month

( ) Yes, more than three times a week

**BLOCK: TESTING HABITS**

53. Have you ever been tested for any STIs before?

( ) Yes, in the private network at diagnostic medicine laboratories

( ) Yes, in the public network via rapid testing

( ) Yes, through an over-the-counter self-test purchased at a pharmacy

( ) Yes, through a self-test distributed by SUS

( ) I have never been tested for STIs

54. What is your main motivation for seeking STI testing?

( ) To know my HIV status

( ) To know my Hepatitis B and C status

( ) To know my Syphilis status

( ) To know my status for other diseases

( ) Not applicable (First test)

( ) Did not respond

55. What is your main motivation for seeking an HIV test?

( ) My partner is HIV+

( ) I don’t know my partner’s serostatus

( ) The condom broke or slipped during sexual intercourse

( ) I believe I have been in a risky situation

( ) I get periodic tests regardless of risk

56. Before volunteering for this study, did you know that SUS provides free testing for HIV and other STIs through testing and counseling centers (CTAs), in some health posts, and in urgent care units?

( ) Did not know

( ) Yes, I knew and have visited these institutions to get tested for STIs.

( ) Yes, I knew, but I have never visited any of these institutions to test for STIs.

57. Did you already know about the oral fluid HIV self-test?

( ) Yes

( ) No

58. Did you know that the oral fluid HIV self-test is already being sold in pharmacies?

( ) Yes

( ) No

59. The self-test is a technology that allows the patient to test themselves at home. If you had access to this technology, how much more frequently would you test for HIV?

( ) I would test less frequently

( ) I would test with the same frequency

( ) I would test more frequently

**BLOCK: DISCRIMINATION/VIOLENCE**

60. Did you seek HIV testing freely?

( ) No, my partner forced me to get tested

( ) No, my partner influenced me to get tested

( ) Yes

( ) Did not respond

Observations:

________________________________________________________________________________

________________________________________________________________________________

61. Have you ever felt discriminated against because of your sexual orientation?

( ) Yes. When?

( ) Childhood

( ) Adolescence

( ) Adulthood

( ) No – Skip to question 64

( ) Did not respond

62. How often has this happened?

( ) Many times

( ) A few times

( ) Rarely

( ) Only once

( ) Not applicable (Did not experience discrimination due to sexual orientation)

( ) Did not respond

63. Who perpetrated the discrimination? (You can mark more than one option):

( ) Father and/or Mother

( ) Other family members

( ) Partner/Spouse

( ) Friends

( ) School/college colleagues

( ) Work colleagues

( ) Health professional

( ) Education institution professional

( ) Work supervisor

( ) Police

( ) Stranger

( ) Other: _________________________

( ) Not applicable (Did not experience discrimination due to sexual orientation)

( ) Did not respond

64. Have you ever suffered any form of physical aggression because of your sexual orientation?

( ) Yes. When?

( ) Childhood

( ) Adolescence

( ) Adulthood

( ) No

( ) Did not respond

65. How often has this happened?

( ) Many times

( ) A few times

( ) Rarely

( ) Only once

( ) Not applicable (Did not suffer physical aggression because of sexual orientation)

( ) Did not respond

66. Who perpetrated this aggression? (You can mark more than one option):

( ) Father and/or Mother

( ) Other family members

( ) Partner/Spouse

( ) Friends

( ) School/college colleagues

( ) Work colleagues

( ) Health professional

( ) Education institution professional

( ) Work supervisor

( ) Police

( ) Stranger

( ) Other: _________________________

( ) Not applicable (Did not experience aggression because of sexual orientation)

( ) Did not respond

**QUESTIONNAIRE CONFIRMATION**

67. How long after a risky exposure (having sex without a condom and sharing needles) should you take a rapid test or self-test for HIV?

( ) Right after the risky behavior

( ) 24 hours after the risky behavior

( ) 15 days after the risky behavior

( ) 30 days after the risky behavior

( ) 3 months after the risky behavior

( ) Don’t know

Observations by the professional:

**Table S4** - HIV DETECT ORAL SELF-TEST SATISFACTION QUESTIONNAIRE (*ECODIAGNÓSTICA*™)

|  | *Statistics* |
| --- | --- |
| How would you rate the tests' instructions? (n=120) |  |
| Very easy | 20 (16.7%) |
| Easy | 94 (78.3%) |
| Difficult | 4 (3.3%) |
| Very Difficult | 2 (1.7%) |
| How would you rate the preparation of the kit components?  (n=118) |  |
| Very easy | 15 (12.7%) |
| Easy | 96 (81.4%) |
| Difficult | 5 (4.2%) |
| Very Difficult | 2 (1.7%) |
| How would you rate the self-test execution? (n=120) |  |
| Very easy | 18 (15.0%) |
| Easy | 100 (83.3%) |
| Difficult | 1 (0.8%) |
| Very Difficult | 1 (0.8%) |
| How would you rate the self-test reading? (n=114) |  |
| Very easy | 32 (28.1%) |
| Easy | 77 (67.5%) |
| Difficult | 5 (4.4%) |
| Very Difficult | 0 (0.0%) |
| How would you rate your reading of the results? (n=114) |  |
| Very well | 37 (32.5%) |
| Well | 74 (64.9%) |
| Bad | 2 (1.8%) |
| Very bad | 1 (0.9%) |
| How has your motivation been affected by this experience?  (n=120) |  |
| I feel more motivated to use the test again | 89 (74.2%) |
| I do not feel differently about performing the test | 27 (22.5%) |
| I feel less motivated to use the test again | 4 (3.3%) |
| Would you use this test again? (n=118) |  |
| No, I would not use it again | 11 (9.3%) |
| Yes, because when compared to traditional testing, the self-test allows for greater privacy and less exposure | 44 (37.3%) |
| Yes, because when compared to traditional tests, the self-test allows greater practicality and accessibility (not having to look for a testing center) | 62 (52.5%) |
| Yes, for another reason | 8 (6.8%) |
| What price would you be willing to pay if the HIVST was sold in pharmacies? (n=119) |  |
| Up to R$ 20 | 35 (29.4%) |
| From R$20 to R$50 | 34 (28.6%) |
| From R$50 to R$80 | 15 (12.6%) |
| From R$80 to R$110 | 21 (17.6%) |
| More than R$110 | 14 (11.8%) |
| How much are you willing to pay for the self-test combo (HIV + HCV + Syphilis) of oral fluid in pharmacies? (n=119) |  |
| Up to R$ 40 | 25 (21.0%) |
| From R$40 to R$80 | 28 (23.5%) |
| From R$80 to R$120 | 34 (28.6%) |
| From R$160 to R$200 | 17 (14.3%) |
| More than de R$200 | 15 (12.6%) |

| If you had access to this technology, how often would you test yourself for HIV? (n=124) |  |
| --- | --- |
| I would test myself less frequently | 4 (3.2%) |
| I would test myself with the same frequency | 31 (25.0%) |
| I would test myself more frequently | 89 (71.8%) |

^n^ Sample size
